# The BSSG rat model of Parkinson’s disease: progressing towards a valid, predictive model of disease

**DOI:** 10.1007/s13167-017-0114-6

**Published:** 2017-09-04

**Authors:** Jackalina M. Van Kampen, Harold A. Robertson

**Affiliations:** 1 Neurodyn Life Sciences, NRC Building, 550 University Ave., Charlottetown, PE C1A 4P3 Canada; 2 Department of Biomedical Sciences, University of Prince Edward Island, Charlottetown, PE C1A 4P3 Canada; 3 Department of Pharmacology, Dalhousie University, Halifax, NS B3H 4R2 Canada

**Keywords:** Parkinson’s disease, Beta-sitosterol beta-d-glucoside (BSSG), α-Synuclein, Nigrostriatal, Dopamine, Non-motor symptoms

## Abstract

**Abstract:**

Parkinson’s disease (PD) is a neurodegenerative disorder, classically considered a movement disorder. A great deal is known about the anatomical connections and neuropathology and pharmacological changes of PD, as they relate to the loss of dopaminergic function and the appearance of cardinal motor symptoms. Our understanding of the role of dopamine in PD has led to the development of effective pharmacological treatments of the motor symptoms in the form of dopamine replacement therapy using levodopa and dopaminergic agonists. Much of the information concerning these drug treatments has been obtained using classical neurotoxic models that mimic dopamine depletion (e.g., 1-methyl-4-phenyl-1,2,3,6-tetrahydropyridine or MPTP, 6-hydroxydopamine, reserpine). However, PD is more than a disorder of the nigrostriatal dopamine pathway. Our understanding of the neuropathology of PD has undergone massive changes, with the discovery that mutations in α-synuclein cause a familial form of PD and that PD pathology may spread, affecting multiple neurotransmitter systems and brain regions. These new developments in our understanding of PD demand that we reconsider our animal models. While classic neurotoxin models have been useful for the development of effective symptomatic treatments for motor manifestations, the paucity of a valid animal model exhibiting the progressive development of multiple key features of PD pathophysiology and phenotype has impeded the search for neuroprotective therapies, capable of slowing or halting disease progression.

**Relevance of the article for predictive, preventive and personalised medicine:**

What characteristics would a good animal model of human PD have? In so much as is possible, a good model would exhibit as many behavioral, anatomical, biochemical, immunological, and pathological changes as are observed in the human condition, developing progressively, with clear, identifiable biomarkers along the way. Here, we review the BSSG rat model of PD, a novel environmental model of PD, with strong construct, face, and predictive validity. This model offers an effective tool for the screening of preventive therapies that may prove to be more predictive of their effects in human patients.

## Introduction

Parkinson’s disease (PD) is a neurodegenerative disorder affecting more than 10 million people, worldwide, with the prevalence of the disease strongly influenced by increasing age [1]. PD has traditionally been classified as a motor disorder characterized by the progressive loss of dopaminergic neurons in the substantia nigra pars compacta (SNc), resulting in significant impairments in the release of dopamine within target sites critical to motor control. The resulting motor symptoms include akinesia, bradykinesia, tremor, and rigidity. Intracellular inclusions containing aggregates of α-synuclein are also key neuropathological hallmarks of PD. The severe loss of striatal dopamine, characteristic of this disease, formed the rationale for dopamine replacement therapy in the form of its precursor, levodopa, which has been the most commonly prescribed treatment for PD motor symptoms since the early 1960s [2]. While levodopa remains the most effective symptomatic treatment, its long-term use is associated with debilitating side-effects, such as dyskinesias and on-off fluctuations. Other forms of treatment include selective dopamine agonists, monoamine oxidase inhibitors, catechol-O-methyltransferase inhibitors, anti-cholinergics, and amantadine, as well as surgical interventions, such as deep brain stimulation and cell transplantation [3, 4]. However, there is currently no effective neuroprotective therapy capable of stopping or slowing the inevitable and insipient progression of the disease.

However, PD can no longer be considered simply a motor disorder resulting from the loss of a discrete population of cells. Rather, it is becoming increasingly apparent that it is a far more complex disease involving the progressive affliction of multiple systems, presenting as both motor and non-motor symptoms [5]. It is this complexity that makes modeling PD such a challenge.

## Challenges in modeling PD

The identification and development of effective neuroprotective therapies has been a key aim of recent PD research. However, many candidates showing promise in preclinical testing have gone on to perform poorly in clinical trials [5–7]. It has been suggested that one reason for these recent failures may be the lack of an appropriate animal model suitable for screening neuroprotective candidates. Ideally, an animal model of PD should: (1) be progressive in nature; (2) closely reflect the disease process, sharing pathological indices with human PD; and (3) reflect the widespread pathology that we now know is associated with the disease in people. To date, animal models of PD have fallen far short of this standard, failing to replicate all of the key features of PD [5].

The classical neurotoxin-based models produced by 6-hydroxydopamine (6-OHDA) and 1-methyl-4-phenyl-1,2,3,6-tetrahydropyridine (MPTP) administration are the most widely used toxic models, having been a staple in PD research for several decades [6, 7] (Table 1). These models trigger mitochondrial complex I inhibition and/or generation of toxic reactive oxygen species, ultimately leading to selective dopaminergic neurodegeneration via apoptosis [8]. While these models do replicate some of the characteristic features of PD, neuronal loss is rapid and not progressive in nature. By contrast, the pathogenic processes underlying PD neuronal cell death involve the complex interplay of a series of events, including mitochondrial dysfunction, oxidative stress, altered protein handling, and inflammation that are interlinked in a way that promotes the generation of a positive feedback loop, initiating a self-perpetuating process that is thought to underlie the progressive nature of the disease [9, 10]. While traditional parkinsonism-inducing neurotoxins do trigger some of these events, they do so in a limited and finite fashion, rather than setting in motion a self-perpetuating cycle of neurodegeneration that progresses independent of the initial triggering event, as occurs in PD. The short time-frame associated with these neurotoxin models is also not reflective of the human condition and the compressed stages preclude the use of these models for furthering our understanding of the pathogenic mechanism of neurodegeneration in PD, which requires a greater temporal delineation of individual events. The acute nature of these models also makes it difficult to screen neuroprotective candidates at time points with greater disease relevance. Indeed, even with early diagnosis, the disease process has already begun. Practical screening of neuroprotective therapies should take place at a time point equivalent to that of a newly diagnosed patient, when early indicators are already evident. Further, the assessment of neuroprotective efficacy is complicated by the remaining presence of the toxin. In order to properly determine that neuroprotection is the result of direct targeting of the disease process rather than an indirect effect of interaction with the toxin, screening must occur in the absence of the toxin. This requires a model that continues to progress long after the initiating event. Thus, the traditional toxin-based animal models are valuable for screening symptomatic treatments, with good predictive validity, but are not ideal for screening neuroprotective approaches [11]. Refinements to these models, such as reduced doses of neurotoxin or intermittent dosing schedules, are currently being explored as ways to generate more protracted models.

**Table 1:** Comparison of the two most widely used neurotoxin models of Parkinson’s disease with the β-sitosterol β-d-glucoside (BSSG model)

Characteristic	6-OHDA	MPTP	BSSG
Progressive	No	No	Yes
Pre-motor changes (e.g., olfaction)	No	No	Yes
Time to significant dopamine cell loss	3–21 days	0.5–21 days	6 months
Loss of substantia nigra pars compacta neurons	Yes	Yes	Yes
Loss of dopamine terminals in striatum	Yes	Yes	Yes
l-dopa-responsive motor deficits	Yes	Yes	Yes
Neuroinflammation	No	No	Yes
Lewy body-like inclusions	No	No	Yes
α-Synuclein accumulation in cortex, hippocampus	No	No	Yes
Late-stage cognitive deficits	No	No	Yes

While classic neurotoxin models, such as 6-OHDA and MPTP, mimic many of the biochemical features of PD, including reduced levels of striatal dopamine and tyrosine hydroxylase, they typically fail to replicate the accumulation of misfolded α-synuclein, the main component of Lewy bodies and Lewy neurites, another cardinal pathological feature of PD [12, 13]. In order for an animal model of PD to have good construct validity, it should display Lewy body-like α-synuclein inclusions. The presence of Lewy bodies and Lewy neurites has become critical in the neuropathological diagnosis of PD and is believed to be a significant pathogenic feature of the disease. Various mutations and polymorphisms in the α-synuclein (SNCA) gene encoding for α-synuclein have been linked to both familial and sporadic PD [14–16], providing a link between the two forms of the disease and emphasizing the significance of this protein in its pathophysiology. Dysregulated α-synuclein contributes to PD pathogenesis in a number of ways, including synaptic and neuritic degeneration, down-regulation of mitochondria complex I activity, ER stress, and proteasomal and lysosomal dysfunction [4, 17]. In view of the importance of α-synuclein in PD pathogenesis, it has become a critical therapeutic target. Various approaches have been proposed, including modulating transcription, blocking aggregation, or enhancing degradation. Effective screening of therapeutic approaches targeting α-synuclein will require a valid animal model of PD displaying Lewy body-like pathology. While various genetic mouse models, targeting the SNCA gene, do display the progressive accumulation of α-synuclein immunoreactive inclusions, they are often lacking a robust loss of dopamine neurons or development of locomotor deficits [18].

While PD has classically been considered a movement disorder, pathology is evident in other non-motor systems resulting in a complex phenotype of both motor and non-motor clinical symptoms [19–21]. Among these are a series of pre-motor or prodromal indications, such as rapid eye movement (REM) sleep disturbances, gastrointestinal dysfunction, and anosmia, which may serve as biomarkers for early diagnosis of PD, preceding motor disturbances by anywhere from 5 to 10 years [20]. These pre-motor symptoms reflect widespread pathology in non-dopaminergic systems, including the brainstem, enteric system, and olfactory bulb [22, 23]. There is now general consensus that neuroprotective approaches will be most efficacious when intervention occurs early in the disease process. Thus, the search for effective disease-modifying therapies goes hand-in-hand with the search for predictive early biomarkers that will permit treatment before significant nigral cell loss has occurred, and it will be important for animal model development to follow suit. A good animal model of PD will be protracted and progressive enough to permit a pre-motor treatment window for screening neuroprotective candidates; one that is marked by a predictive biomarker similar to that found in human cases of PD. Another major non-motor feature of PD is cognitive impairment, which occurs in 30–40% of cases [24, 25], although the cumulative prevalence has been estimated at over 80% [26]. PD dementia is characterized by predominant deficits in executive, visuospatial functions, attention, and memory and is associated with hippocampal and cortical Lewy pathology and cholinergic dysfunction [27, 28]. There is, currently, no valid rodent model of PD dementia.

Braak staging of PD [29] describes the presence of α-synuclein aggregates in different brain regions, appearing in six stages, with pathology originating in the region of the olfactory bulb and/or dorsal motor nucleus of the vagus and spreading to nigrostriatal regions, and then on to hippocampal and cortical regions, coincident with the manifestation of pre-motor, motor, and cognitive/emotional symptoms, respectively. This spread in pathology may result, at least in part, from the notion that α-synuclein, particularly oligomeric forms, may self-propagate and spread progressively between interconnected brain regions via a cell-to-cell transmission mechanism [30–32]. Evidence for this prion-like behavior first came from fetal cell transplant studies showing the appearance of Lewy bodies in host cells more than ten years following transplant [33], with a similar effect found in experimental rodent studies [34, 35]. This propagation of PD pathology may represent a potential target for neuroprotection, one that will require an animal model displaying a similar topographical spread of α-synuclein pathology.

## The cycad hypothesis

**Fig. 1:**
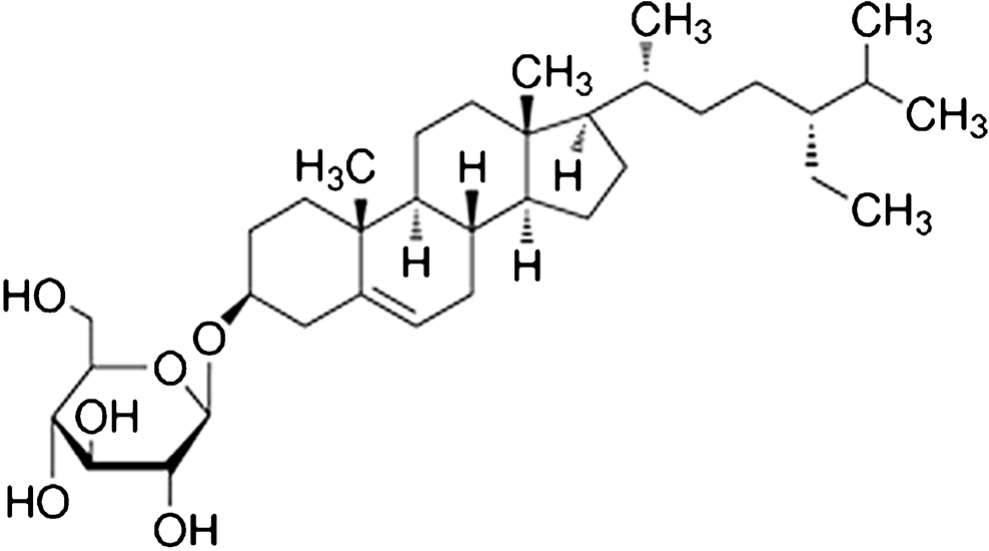
Chemical structure of BSSG

The amyotrophic lateral sclerosis (ALS)/parkinsonism-dementia complex (ALS-PDC) of Guam displays a diverse phenotypic expression with characteristic features of classical ALS, parkinsonism, and dementia [36–38]. During the occupation of the island of Guam by Japan between 1941 and 1944, the indigenous Chamorro people lived on a subsistence diet that was comprised, in large part, of flour made from washed seeds of the plant, *Cycas micronesica* (cycad), known to contain numerous toxins. By 1953, the prevalence of an ALS phenotype was 100 times that of other regions. In the village of Umatac, 25% of adult deaths were due to ALS-PDC. Over the past 50 years, the incidence and prevalence of ALS-PDC has declined significantly, suggesting a strong causal role for environmental factors. It has been suggested that the dietary consumption of cycad seeds is an underlying cause of ALS-PDC. When mice (CD-1, C57BL/6) are fed pellets made from cycad flour, they develop progressive motor and cognitive dysfunctions along with histopathological correlates, including neurodegeneration in the neocortex, hippocampus, substantia nigra, olfactory bulb, and spinal cord [39, 40]. In rats (SD), cycad consumption is similarly neurotoxic, triggering a progressive neurodegeneration, with behavioral, biochemical, and histological hallmarks that are characteristic of parkinsonism, rather than ALS [41]. Indeed, no loss of motor neurons is observed in the spinal cord of rats following chronic cycad consumption. It remains unclear why these species differences in phenotypic expression occur. However, it is not the first example of species differences in PD models. MPTP is perhaps the most obvious example, with peripheral exposure to MPTP triggering profound loss of dopaminergic neurons in mice and even monkeys, while rats remain relatively resistant to systemic exposure [42, 43]. It may require a further understanding of the mechanism of action before these species differences can be explained. Washed cycad flour has been found to contain a variety of water-insoluble neurotoxic candidates, with the most toxic component being identified as beta-sitosterol beta-d-glucoside (BSSG) [39, 44] (Fig. 1). Cell culture and organotypic slice culture studies have confirmed the toxicity of BSSG in all cell types affected in ALS-PDC. In mice, BSSG consumption led to the progressive development of an ALS-PDC phenotype, with a clearly delineated temporal sequence of behavioral and pathological changes in the CNS similar to that previously observed with cycad flour [45]. As with cycad, consumption of BSSG by rats triggers a progressive neurodegeneration that presents as primarily a parkinsonian phenotype, with no evidence of spinal cord pathology, and replicates multiple features of the human disease [41, 46, 47].

## Generating the BSSG model of PD

Stigmasterol (95% purity) is purchased from Sigma-Aldrich Co. (Oakville, ON, Canada) to use as the starting material for the synthesis of BSSG. Stigmasterol is converted to stigmasterol tosylate, which is treated with methanol and pyridine to yield stigmasterol methyl ether. Hydrogenation of stigmasterol methyl ether is accomplished in ethanol using 5% palladium on carbon as a catalyst. Finally, ß-sitosterol is produced in a reaction with tosic acid in water and dioxane at 80 °C. BSSG is characterized using NMR (^1^H and ^13^C) and has, in previous syntheses, been determined to be 95–97% pure. The BSSG is then incorporated into flour pellets (3 mg/pellet), approximately 1–2 cm in diameter. The pellets are mixed, colored for identification (flour control vs BSSG), and flavored (vanilla or banana) to be palatable to rats. Pellets are then dried overnight in an oven, individually sealed, and frozen for storage.

**Fig. 2:**
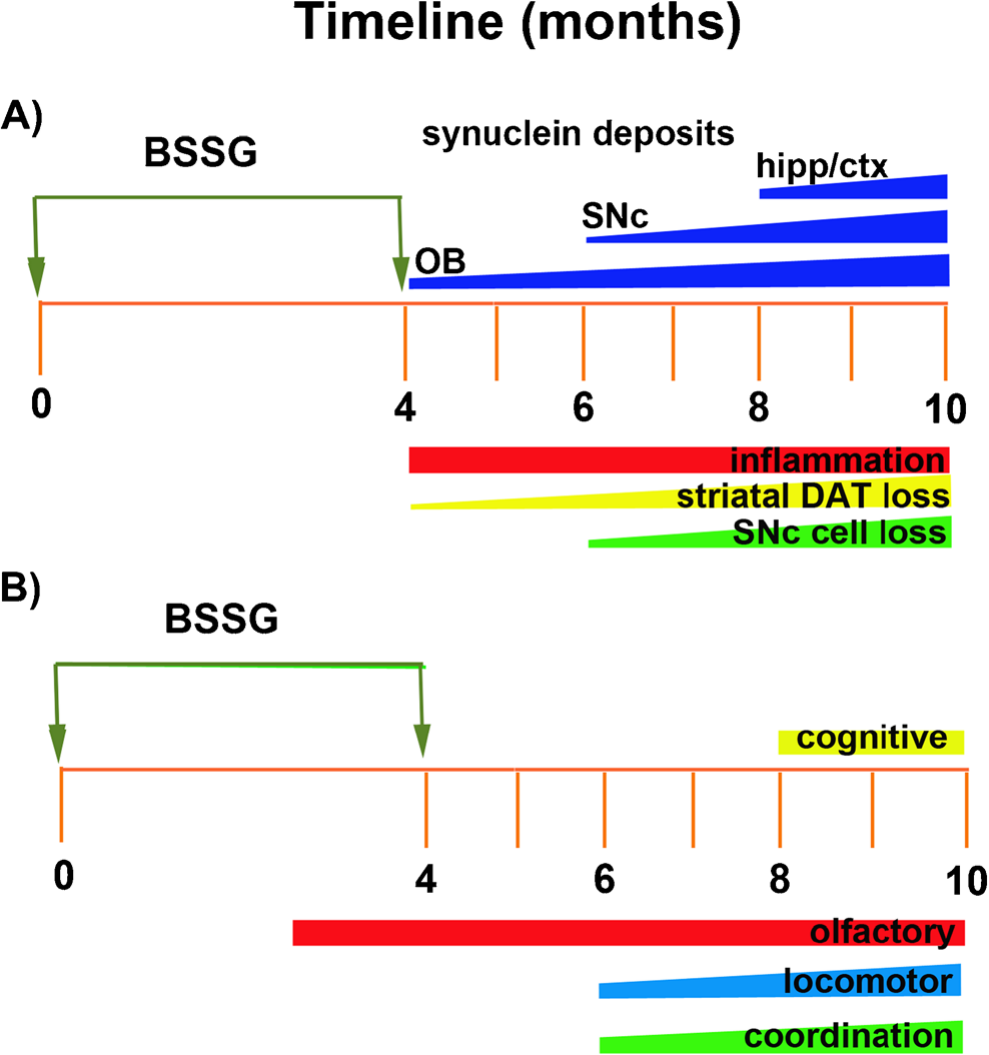
Timeline of key (**a**) histological and (**b**) behavioral events in the phenotypic development of the BSSG model of PD. hipp—hippocampus; ctx—cortex; SNc—substantia nigra pars compacta; OB—olfactory bulb; DAT—dopamine transporter

Male Sprague Dawley rats (aged 3–4 months) are placed on a feeding schedule that restricts food access 8–12 h prior to pellet consumption in order to minimize gastrointestinal competition and maximize absorption. Other strains of rat have not yet been tested. This approach generates a more robust and earlier phenotype than feeding BSSG in the context of ad libitum food access (*data not published*). Ad libitum access to standard rat chow is reinstated 2–3 h following pellet consumption. Animals are fed BSSG pellets (3 mg) once per day, 5 days per week, for 16 weeks. In developing the parameters for the BSSG model, it was found that this 2-day break in the feeding schedule resulted in faster and more profound pathology development (*data not published*). Following 16 weeks of feeding, BSSG exposure is terminated and the phenotype permitted to develop, in the absence of the toxin, for another 4–6 months, depending on the target of interest. A timeline of events is depicted in Fig. 2.

## BSSG model of PD

### Motor features

The BSSG model of PD is characterized by the progressive loss of dopaminergic neurons of the nigrostriatal pathway, resulting in akinesia, bradykinesia, and deficits in locomotor coordination [46, 47]. At the time BSSG exposure is terminated, there is not yet any significant loss of dopamine neurons in the SNc. However, nigral cell loss develops progressively over the ensuing months, reaching 75–80% cell loss at 10 months following initial BSSG exposure. This loss of nigrostriatal neurons occurs in an asymmetric pattern, with greater pathology observed in one hemisphere. This asymmetry is at its greatest at 6 months following initial BSSG exposure and wanes thereafter, as the degeneration becomes more bilateral. This asymmetry is also reflected in the loss of dopaminergic terminals in the striatum, as measured by dopamine transporter (DAT) immunodensity. The loss of striatal innervation by dopaminergic neurons precedes SNc cell loss, reaching significance by 4 months following initial BSSG exposure. However, a significant asymmetry in terminal loss is observed at this time point; one that resolves itself by 8 months. No locomotor deficits are apparent at the time BSSG feeding is completed, consistent with the lack of SNc cell loss. However, the asymmetric loss of striatal innervation results in locomotor asymmetry upon methamphetamine challenge, in the form of rotations, which are no longer present 10 months following initial BSSG exposure. This initial asymmetric presentation is consistent with Hoehn and Yahr Stage 1, which describes an initial unilateral onset that progresses to become bilateral by Stage 2 [48, 49].

In the BSSG model of PD, the progressive degeneration of nigrostriatal neurons results in the gradual development of locomotor deficits. As mentioned, no significant locomotor deficits are apparent at the time BSSG feeding is completed, with the exception of methamphetamine-induced rotations. In an open field test, locomotor activity is significantly reduced, beginning at 6 months following initial BSSG exposure, and continues to decline over the course of the subsequent 4 months. No testing has yet been done beyond this time point. Locomotor coordination is also affected in these animals. On a horizontal ladder test, BSSG-fed animals displayed significantly more foot slips, beginning at 6 months, and worsening over time. Impairments in skilled reaching have also been observed in the BSSG model [47], as assessed by the staircase step test [50], a sensitive measure of motor system damage [51, 52]. In order for these locomotor deficits to properly model human PD, they must be responsive to levodopa, the same way PD motor symptoms are responsive. This dopamine replacement therapy also confirms the involvement of dopamine depletion as the causative factor. In the BSSG model of PD, all locomotor deficits are completely reversed by the anti-parkinsonian drug, levodopa/carbidopa, further validating these behavioral indices as appropriate measures of a parkinsonian phenotype [46].

### Prodromal features

There is a general consensus that neuroprotective therapies for PD will be most effective with an early intervention. Ideally, this would occur prior to significant degeneration of the motor system and resulting motor impairments [53, 54]. Thus, the identification of early pre-motor biomarkers has been a critical aspect of PD research. To date, a number of early biomarkers have been identified. Those with the greatest predictive capability include REM sleep disturbances, gastrointestinal (GI) issues, such as constipation, and olfactory deficits [54, 55]. The majority of PD patients present with anosmia, with prevalence estimates as high as 90% [56], and these deficits can predate diagnosis by as much as a decade [57]. This is consistent with the postulation by Braak that synuclein pathology occurs first in olfactory structures, prior to its appearance in motor structures, such as the SNc [29, 58]. Effective screening of neuroprotective therapies will require animal models with a similar prodromal phase, offering comparable pre-motor biomarkers.

Following BSSG intoxication in rats, olfactory dysfunction is the earliest behavioral deficit observed, occurring as early as 3 months following initial BSSG exposure, and preceding significant motor impairments by 3 months. These olfactory deficits not only appear early but persist throughout phenotypic development of the model, with no indications of recovery. This olfactory deficit occurs in conjunction with the early appearance of proteinase K-resistant α-synuclein aggregates and microgliosis in the olfactory bulb, while other regions of the CNS remained relatively unaffected at this early time point [46]. This is consistent with olfactory deficits known to occur in human cases of ALS-PDC in Guam [59] and olfactory degeneration associated with cycad consumption [39, 60]. Cycad consumption in rats is associated with the early, pre-motor appearance of sleep disturbances in the form of disrupted REM sleep patterns and hypersomnolence, very similar to that observed in human cases of PD [61]. Early indications of GI pathology following BSSG exposure are currently being explored. Certainly, components of cycad are known to irritate the GI tract [62, 63], and chronic cycad consumption in mice has been shown to trigger apoptosis in the gut [64]. As GI disturbances are an early pre-motor feature of PD, and pathology in the gut may play a role in driving PD pathogenesis in the brain [65], it will be very interesting to further explore these GI changes and their potential role in the development of the BSSG model.

### Cognitive deficits

Another key non-motor feature of PD is the development of cognitive deficits. Although estimates vary, the majority of patients with PD will develop dementia should they survive for more than 10 years following diagnosis [25, 66]. Unlike the prodromal features described above, PD dementia typically occurs well after an established diagnosis of PD. While the mechanisms underlying PD dementia are still being explored, the spread of pathology to other regions and systems has been implicated as a key factor. According to the Braak’s hypothesis, the appearance of synuclein pathology in motor systems is then followed, in later stages, by their appearance in higher-order cortical and mesocortical regions [29]. In some cases of cognitive decline, tau and β-amyloid pathology have also been implicated, along with reductions in cholinergic neurotransmission and synaptic atrophy [27, 66, 67]. Currently, there is no good rodent model of PD dementia. However, the BSSG model of PD features late-stage cognitive deficits, in the form of impairments in both reference and spatial working memory, as assessed by the eight-arm radial arm maze. Short-term working memory deficits, assessed by the spontaneous alternation T-maze, are also evident in the late stage of phenotypic development, months following the development of locomotor deficits, but are not apparent in the pre-motor stage. These cognitive deficits appear at a time when synuclein pathology is detectable in hippocampal and cortical regions of the brain, along with markers of inflammation and a significant loss of synaptic density in the hippocampus and prefrontal cortex. This is consistent with the co-occurrence of parkinsonism and dementia in the affected Chamorros of Guam, purportedly linked to excessive cycad consumption [68, 69] as well as the late-stage appearance of cognitive dysfunctions observed in conjunction with neuronal cell death in the regions of cortex and hippocampus [39]. Thus, the BSSG model of PD also has potential as a rodent model of PD dementia that may be useful for examining disease development and screening therapeutics. Studies are currently underway to determine whether deficits in executive function, typical of PD dementia [67, 70], are also evident in the BSSG model and what other forms of pathology may be underlying these deficits.

### Synuclein pathology

Lewy bodies are a key pathological feature of PD and contain large amounts of α-synuclein aggregates. Under pathological conditions, α-synuclein is capable of generating β-sheet structures, which form the basis for mature Lewy bodies and neurites. Aberrant α-synuclein accumulation, including both mature fibrils and intermediate oligomeric forms, contributes to PD pathogenesis in a number of different ways. Aggregation of α-synuclein has been shown to disrupt neurotransmitter release [71], trigger synaptic degeneration [72], undermine cytoskeletal integrity [73], impair proteasomal and lysosomal clearance of aberrant proteins [74], trigger mitochondrial impairments [75], promote Golgi/endoplasmic reticulum stress [76], induce inflammatory reactions in glial cells [77], and promote further abnormal intracellular protein aggregation [78]. Thus, abnormal α-synuclein aggregates can trigger multiple deleterious events, many of which can further potentiate neurodegeneration, possibly contributing to the progressive nature of the disease.

**Fig. 3:**
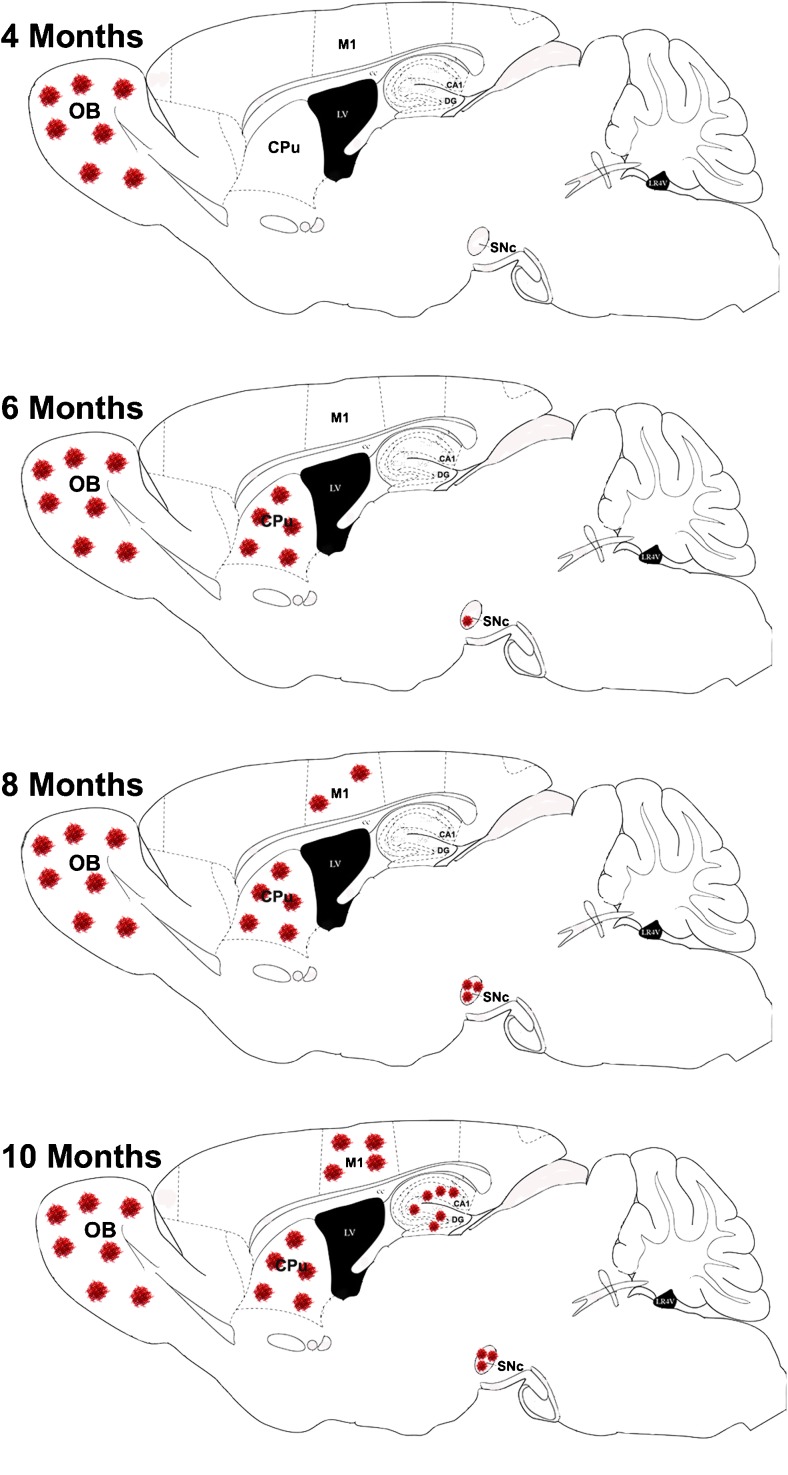
Diagram depicting the topographical appearance of α-synuclein aggregates over time in the BSSG model of PD. OB—olfactory bulb; CPu—caudate putamen (striatum); M1—motor cortex; SNc—substantia nigra pars compacta; CA1—Cornu Ammonis area 1; DG—dentate gyrus

Perhaps the most intriguing feature of the BSSG model of PD is the progressive topographical spread of proteinase K-resistant α-synuclein aggregates over time that coincides, temporally, with the appearance of pre-motor, motor, and cognitive deficits in these animals [46] (Fig. 3). A subset of these aggregates was positive for Thioflavin S, indicating a β-sheet structure. It has been demonstrated, in vitro, that sterol glucosides derived from cycad can directly promote the aggregation of α-synuclein and enhance the resulting cytotoxicity [79]. This is consistent with the appearance of α-synuclein pathology in the brains of Guamanian ALS-PDC cases [36, 38, 80, 81] and the appearance of synuclein inclusions in the brains of rats following the chronic consumption of cycad [41] and its toxic component, BSSG [46, 47]. This unique regional progression of synuclein pathology is similar to that reported in PD patients [82, 83]. Indeed, the development of synuclein pathology in the BSSG model closely resembles the Braak’s staging hypothesis of PD, which describes the appearance of inclusion body formations in a similar topographical stepwise fashion, with the type and severity of symptoms correlated with pathology progression through the Braak stages [29, 82]. This hypothesis supports the notion of PD as a prion disorder, in which misfolded proteins are capable of spreading and seeding neighboring CNS regions. Secreted α-synuclein aggregates are taken up by neighboring cells and promote fibrilization of endogenous synuclein [84, 85]. This is intriguingly demonstrated in fetal transplant studies, in which evidence of α-synuclein-positive, Lewy body-like inclusions was detected in dopaminergic cells grafted into the putamen of human PD patients [33, 86, 87] and experimental rats [34]. Although there is not a general consensus on the transmission of pathology [88] and the mechanism responsible is not yet known, understanding this process may be critical for slowing or halting disease progression. Therapies targeting the spread of misfolded protein will require a valid progressive animal model, displaying the gradual region-by-region development of synucleinopathy that has been observed in PD.

### Mechanism of action

Parkinson’s disease is caused by a complex interaction between genetic and environmental processes. While the etiology of familial PD has provided important molecular pathways and processes involved in the neuropathology of this disorder, a common mechanism of action for sporadic PD remains largely unknown. The fundamental issues in PD are, first, loss of dopaminergic (DA) neurons in the substantia nigra (SN), and second, the presence of protein aggregates (Lewy bodies) involving SNCA in the remaining DA neurons [89]. A number of biological processes that contribute to the pathogenesis of PD have been identified, including defects in mitochondrial function [90], oxidative stress [91], and protein aggregation [92]. However, detailed insights into the molecular mechanisms underlying these processes, and how they interact with each other, are essentially lacking. In many studies exploring PD pathogenesis, familial PD genes or toxins known to produce symptoms in animals and man have served as starting point for model development. A good example of a toxin with effects first described in man and subsequently shown to produce neuropathology in man similar to sporadic PD is MPTP. Likewise some of the strength of the BSSG model lies in its roots in human neuropathology and, in particular, in the almost perfect replication of the changes observed in sporadic PD.

The development of the BSSG model as described here is another good example of a toxin initially described as producing a constellation of human neurological disorders in Guam (ALS/parkinsonism-dementia complex or ALS-PDC). Careful study of the alcohol extract revealed the presence of several sterol glucosides including BSSG [44]. Although the mechanisms underlying the pathology remain unknown, alterations in lipid homeostasis appear to be a central factor. Maintenance of lipid homeostasis is increasingly recognized as a crucial factor for normal neuronal function. Modulation of cerebral lipid metabolism or transport may be linked to neurodegenerative pathways in PD [93]. Of particular interest is the finding that α-synuclein is a lipid-binding protein and that it deposits with lipids associated with Lewy bodies and neuromelanin in PD tissues [94–96]. Dietary plant sterols are structurally very similar to cholesterol and readily cross the blood-brain barrier and accumulate in the brain [97] where they act as liver X receptor ligands to regulate cholesterol homeostasis [97, 98]. Liver X receptor β (LXRβ) is expressed in microglia and astroglia of the SNc and has been found to play an important role in dopaminergic neuron survival [98–100]. Disruptions in LXRβ signaling by BSSG could lead to alterations in cholesterol homeostasis, activation of microglia, accumulation of synuclein aggregates, and, ultimately, nigral cell loss. Efforts are currently underway to elucidate this potential mechanism of action. The BSSG model is potentially at the center of developments in regulation of liver metabolism (liver XR), Gaucher disease (mutations in the glucocerebrosidase gene (GBA1)), and their role in increased risk for α-synuclein aggregation disorders (“synucleinopathies”), which include PD and dementia with Lewy bodies (DLB). Other findings also implicate the role of lipids. Recent studies have shown that glucocerebrosidase can act as a regulator of sterol glucoside metabolism and β-sitosterylglucoside can be found in vertebrate brain [101, 102]. Finally, evidence comes from studies of a natural product, squalamine, which has anticancer and antiviral activities and which dramatically affects α-synuclein aggregation via inhibition of the interaction between α-synuclein oligomers and lipid membranes [103]. The stage is therefore set to develop a mechanism of action of BSSG that will advance the search for targets that might slow or even halt progression of PD and related disorders.

## Benefits of the BSSG model

The BSSG model of PD has many benefits. Probably its most valuable feature is its progressive nature. This will be critical for screening neuroprotective therapies. Rather than targeting acute responses to the initiating factor, effective therapeutic interventions will need to target the self-perpetuating cascade of pathological events that continues long after the initial insult occurs. Effective screening of such therapies will require an animal model capable of recapitulating disease progression in the absence of the initiating event/toxin. Further, the protracted nature of the model and the appearance of pre-motor indices make it possible for such neuroprotective therapies to be tested at a time point in model development that is analogous to the prodromal phase of human PD. With ongoing development of early biomarkers, diagnosis will occur earlier in disease development, permitting early and, thus, more effective neuroprotective interventions. This model is also the most comprehensive rodent model of PD, recapitulating a series of pathological events and phenotypic features characteristic of human PD. Often, current models fail to display one or more key features, such as acute toxin models, which typically display nigral cell loss but no Lewy body-like inclusions or α-synuclein transgenic models, which often display synucleinopathy but minimal nigral cell loss or locomotor phenotype. Since the pathophysiology of PD involves an interplay of various events that amplify each other, targeting one event in the absence of others may not provide a valid assessment of how a therapy might perform in the context of human PD. The apparent topographical spread of synuclein pathology is also uniquely suited to screening therapies aimed at slowing or blocking synuclein uptake and “seeding” of neighboring cells. Along the same lines, much attention has recently focussed on the role of the gut in driving PD pathology in the brain, a notion consistent with Braak’s staging hypothesis. While the role of the gut/brain axis in PD is still being explored, this research could greatly benefit from an animal model initiated in the gut, as is the case with the BSSG model. Finally, there is no good model of PD dementia. Current therapies for treating dementia in PD are screened in models of Alzheimer’s disease or acute models of cognitive impairment, which fail to replicate the ongoing pathological events associated with PD. Given the influence of synuclein aggregates on synaptic function, neurotransmitter release, and membrane permeability, it may be important to screen PD dementia therapies in the context of a PD phenotype. Cycad and its toxic component, BSSG, have been used to model neurodegenerative disease in rodents by a number of groups for several years [39, 41, 45–47, 104], and the BSSG model of PD is currently undergoing an independent multi-site validation, with added value components that will help further our understanding of this multi-faceted model. Of course, the model does require considerable time to fully develop, which can translate into high animal housing costs. This is a natural pitfall of any model that truly replicates the progressive nature of PD, a disease that takes several years to manifest. However, with continued research, the description of early biomarkers will offer experimental endpoints that may preclude the need for full phenotypic development. While this model may not be cost-effective for early screening, it may provide an intermediary step that could prevent costly failed clinical trials. Overall, the BSSG model of PD provides construct, face, and predictive validity that will be valuable for the screening of potential therapeutic and neuroprotective therapies for the treatment of PD.
